# Immuno-OpenPET: a novel approach for early diagnosis and image-guided surgery for small resectable pancreatic cancer

**DOI:** 10.1038/s41598-020-61056-5

**Published:** 2020-03-10

**Authors:** Yukie Yoshii, Hideaki Tashima, Yuma Iwao, Eiji Yoshida, Hidekatsu Wakizaka, Go Akamatsu, Taiga Yamaya, Hiroki Matsumoto, Mitsuyoshi Yoshimoto, Chika Igarashi, Fukiko Hihara, Tomoko Tachibana, Ming-Rong Zhang, Kotaro Nagatsu, Aya Sugyo, Atsushi B. Tsuji, Tatsuya Higashi

**Affiliations:** 1 National Institute of Radiological Sciences, National Institutes for Quantum and Radiological Science and Technology, Chiba, 263-8555 Japan; 2 Nihon Medi-Physics Co., Ltd., Tokyo, 136-0075 Japan; 3 Division of Functional Imaging, National Cancer Center Hospital East, Kashiwa, Chiba 277-8577 Japan

**Keywords:** Cancer imaging, Surgical oncology

## Abstract

Pancreatic cancer (PC) has a poor prognosis owing to difficulties in the diagnosis of resectable PC at early stages. Several clinical studies have indicated that the detection and surgery of small resectable PC (<1 cm) can significantly improve survival; however, imaging diagnosis and accurate resection of small PC remain challenging. Here, we report the feasibility of “immuno-OpenPET” as a novel approach enabling not only early diagnosis but also image-guided surgery, using a small (<1 cm) resectable PC orthotopic xenograft mouse model. For immuno-OpenPET, we utilized our original OpenPET system, which enables high-resolution positron emission tomography (PET) imaging with depth-of-interaction detectors, as well as real-time image-guided surgery, by arranging the detectors to create an open space for surgery and accelerating the image reconstruction process by graphics processing units. For immuno-OpenPET, ^64^Cu-labeled anti-epidermal growth factor receptor antibody cetuximab was intraperitoneally administered into mice. It clearly identified PC tumors ≥3 mm. In contrast, neither OpenPET with intravenous-administered ^64^Cu-cetuximab nor intraperitoneal/intravenous-administered ^18^F-FDG (a traditional PET probe) could detect PC in this model. Immuno-OpenPET-guided surgery accurately resected small PC in mice and achieved significantly prolonged survival. This technology could provide a novel diagnostic and therapeutic strategy for small resectable PC to improve patient survival.

## Introduction

Pancreatic cancer (PC) has a poor prognosis with an overall 5-year survival rate below 10%^1–4^, which is due to the difficulty of PC detection at early stages when classified as resectable disease^5^. Several clinical studies have indicated that surgery of small resectable PC tumors <1 cm can significantly improve the prognosis compared to that of larger lesions, although it’s hard to detect PC at early stages^6–8^. To improve this situation, clinical studies to develop new plasma biomarkers, such as proteins, miRNAs and metabolites, are ongoing worldwide, which will achieve screening patients at early-stage PC^9–14^. However, imaging diagnosis for localization of small resectable PC using current modalities such as computed tomography, magnetic resonance imaging, endoscopic ultrasound, and ^18^F-fluorodeoxyglucose (^18^F-FDG) positron emission tomography (PET) remains challenging due to the limited resolution and low contrast^13^. Therefore, precise imaging methods are needed to improve the diagnosis for suspected cases of PC after screening with new plasma biomarkers but diagnosed as negative or ambiguous based on current imaging modalities. Moreover, the identification of small PC lesions within the pancreas during surgery is also critical for accurate resection.

Recently, we developed the world’s first open-type PET system called “OpenPET”^15–17^ (Supplementary Fig. S1, Table S1). This system equips depth-of-interaction detectors, which can provide high-resolution imaging (approximately 2 mm). The OpenPET arranges the detectors to create a sufficient open space for conducting surgical procedures. It also adopts a rapid image reconstitution system with graphics processing units, which can provide continual images in real time by updating in cycles of <1 s while accumulating data. These distinguished properties of this PET system facilitate diagnosis as well as image-guided surgery with high resolution. This system is potentially adoptable for endoscopic surgery and robotic surgery as well as for general surgery. We previously demonstrated that OpenPET with intraperitoneal administration of anti-epidermal growth factor receptor (EGFR) antibody cetuximab labeled with ^64^Cu (β^+^ decay 0.653 MeV, 17.4%; β^−^ decay, 0.574 MeV, 40%; electron capture, 42.6%) was useful for the diagnosis and guided surgery of gastrointestinal cancer-derived tumors in the mouse peritoneal cavity, and intraperitoneal administration of this probe led to their higher accumulation than intravenous injection^18^. Subsequently^19^, we demonstrated the potential of intraperitoneal administration of ^64^Cu-cetuximab as an adjuvant treatment for PC, since EGFR (the target of cetuximab) is overexpressed in up to 90% of PCs^20,21^, and the β^−^ particles and high-linear energy transfer Auger electrons from ^64^Cu can effectively damage tumor cells. Indeed, intraperitoneal-administrated ^64^Cu-cetuximab was efficiently delivered to PC within the pancreas as well as to liver metastases and peritoneal dissemination, and was effective as an adjuvant treatment after PC surgery in a clinically relevant orthotopic PC xenografted mouse model^19^.

Based on this background, we hypothesized that OpenPET with intraperitoneal-administered ^64^Cu-cetuximab (hereafter immuno-OpenPET) would be useful for early diagnosis in suspected PC cases and applicable to image-guided surgery for accurate resection. Here, we demonstrated the feasibility of immuno-OpenPET for early diagnosis of PC using a mouse model with a small resectable human PC orthotopic xenograft (<1 cm) and the efficacy of image-guided surgery with immuno-OpenPET in this model.

## Materials and Methods

### Preparation of ^64^Cu-labeled cetuximab

Cetuximab was obtained from Merck Serono. ^64^Cu was produced and purified as previously described^22^. Cetuximab was ^64^Cu-labeled using 3,6,9,15-tetraazabicyclo[9.3.1]pentadeca-1(15),11,13-triene-3,6,9-triacetic acid (PCTA) as the chelator, which was previously found to result in a high radiolabeling yield and *in vitro* serum stability^18,23^. ^64^Cu-PCTA-cetuximab was prepared according to previously reported methods^18^ with specific activity ranging from 1.1 to 1.7 GBq/mg. The injected protein dose of ^64^Cu-PCTA-cetuximab was adjusted to 20 µg per mouse by adding an unlabeled antibody as reported previously^18^.

### Cell culture and small resectable orthotopic xenograft model

Human PC xPA-1 cells expressing red fluorescent protein in the cytoplasm and green fluorescent protein in the nucleus (xPA-1-dual color, xPA-1-DC) (AntiCancer) with EGFR overexpression^19^ were used for establishment of the mouse model. xPA-1-DC cells were cultured in RPMI-1640 medium (Wako) supplemented with 10% fetal bovine serum in a humidified atmosphere of 95% air and 5% CO_2_ at 37 °C.

Six-week-old female BALB/c nude mice were obtained from Japan SLC. The animals were maintained and handled in accordance with the recommendations of National Institute of Health and the institutional guidelines of National Institute of Radiological Sciences. The study protocols were approved by the Animal Ethics Committee of National Institute of Radiological Sciences. To establish the small resectable orthotopic xenograft model, pieces of tumor tissues were prepared from mice with tumor xenografts and implanted into other mice according to the following procedures. First, xPA-1-DC cells (5 × 10^6^) in 25 µL of RPMI-1640 medium mixed with 25 µL of ice-cold extracellular matrix (Matrigel matrix, BD Biosciences) were injected into the pancreatic tail through the incision. One week later, the developed xPA-1-DC tumors were isolated and minced with a razor to obtain small (approximately 1 mm^3^) pieces. One tumor piece was gently inserted at the tip of a 21-gauge needle filled with 20 µL of Matrigel matrix, and slowly injected into the tail of the pancreas to avoid leakage and facilitate engraftment (Supplementary Fig. S2). To determine the optimal timing for this clinically relevant model of small resectable PC, the mice were sacrificed at 1, 2, and 3 weeks after tumor implantation, and the tumor size and weight were measured. Levels of traditional plasma biomarkers (CA19-9 and CEA) were also observed for comparison (n = 5 per time point). Tumor implantation and observation of tumor development were performed with the aid of a stereoscopic fluorescence microscope (MZ16F, Leica). The signals from red fluorescent protein were measured to identify tumors. From these observations, we chose to carry out immuno-OpenPET at 2 weeks after tumor implantation because tumor lesions <1 cm were formed in all observed mice at this time point.

### Immuno-OpenPET imaging for early diagnosis of PC

The small resectable orthotopic xPA-1-DC xenograft mouse model at 2 weeks after tumor implantation was used to examine the feasibility of immuno-OpenPET imaging for the early diagnosis of PC. ^64^Cu-PCTA-cetuximab (7.4 MBq/mouse) was intraperitoneally administered to the mice, and PET images were obtained 24 h later with the small OpenPET system (n = 6). This system was developed for use in small animal experiments and has a spatial resolution of approximately 2 mm^15,16^ (Supplementary Fig. S1, left, Supplementary Table S1). At 5 min after small OpenPET imaging, the PET images were obtained for the same mice with the human-sized OpenPET system, which was developed for human use with an equivalent spatial resolution to that of the small system^24^ (Supplementary Fig. S1, right, Supplementary Table S1). For comparison, intravenous-administered ^64^Cu-PCTA-cetuximab (7.4 MBq/mouse, n = 6) and intraperitoneal or intravenous-administered ^18^F-FDG (1.85 MBq/mouse, n = 4 per group) were also tested in the same model with the small OpenPET system. OpenPET imaging was performed 24 h after intravenous-administered ^64^Cu-PCTA-cetuximab and at 1 h after intraperitoneal or intravenous-administered ^18^F-FDG. The injected dose and timing of ^64^Cu-PCTA-cetuximab and ^18^F-FDG were determined based on previous reports^18,25^. The imaging acquisition and analysis were performed according to previously described procedures^18^ with a 10-min data acquisition. After the OpenPET imaging, laparotomy and observation with the stereoscopic fluorescence microscope were performed to determine tumor localization. To characterize the tumor uptake of ^64^Cu-PCTA-cetuximab and ^18^F-FDG, xPA-1-DC orthotopic xenografts were isolated immediately after the OpenPET imaging. Tumors were weighed and radioactivity levels were measured with a γ-counter (1480 Wizard 3; PerkinElmer). The percentage of injected dose per gram (%ID/g) was evaluated as reported previously^19^.

### Immuno-OpenPET-guided surgery

The effectiveness of image-guided surgery with immuno-OpenPET was evaluated in mice bearing the small resectable orthotopic xPA-1-DC xenografts 2 weeks after tumor implantation. The model mice were randomized into two groups: image-guided surgery with immuno-OpenPET (immuno-OpenPET surgery) and non-treated (control) groups (n = 10/group) (day 0). In the immuno-OpenPET surgery group, ^64^Cu-PCTA-cetuximab (7.4 MBq/mouse) was intraperitoneally administered to the mice at day 0 and then OpenPET-guided surgery was conducted 24 h later with the small OpenPET system as previously described^18^. Before beginning the surgery, real-time OpenPET images were obtained to identify the tumor locations, and then the abdominal wall and skin were cut to exteriorize the pancreas. While checking the position of the tumor within the pancreas in real time using the OpenPET system, the part of the pancreas most proximal to the tumor was ligated with a clamp to prevent bleeding, and then partial pancreatectomy was performed to resect the pancreatic tail containing PC. The pancreas was then returned to the abdomen, and the peritoneum and skin were closed using surgical sutures.

The resected pancreas was examined with a stereoscopic fluorescence microscope to ensure the presence of the tumors in the surgical specimens. The same surgical procedures with partial pancreas resection were conducted in five tumor-free mice to observe general conditions and body weight change, and to confirm the absence of postoperative adverse effects. Considering that such small PC lesions cannot be detected with current clinical detection systems in practice, the control group was not given any treatment. For the immuno-OpenPET surgery and control groups, mortality of the mice was monitored until day 72. The mice were sacrificed when reaching a humane endpoint, which was defined as noticeable extension of the abdomen, development of ascites, or body weight loss >20%^26,27^.

### Statistical analysis

Data are expressed as the mean and standard deviation. *P* values were calculated using one-way analysis of variance with the Tukey-Kramer multiple comparison post-hoc test. Differences in survival were evaluated by the log-rank test. *P* values < 0.05 were considered to be statistically significant.

## Results

### Establishment of the small resectable orthotopic PC xenograft model

**Figure 1:**
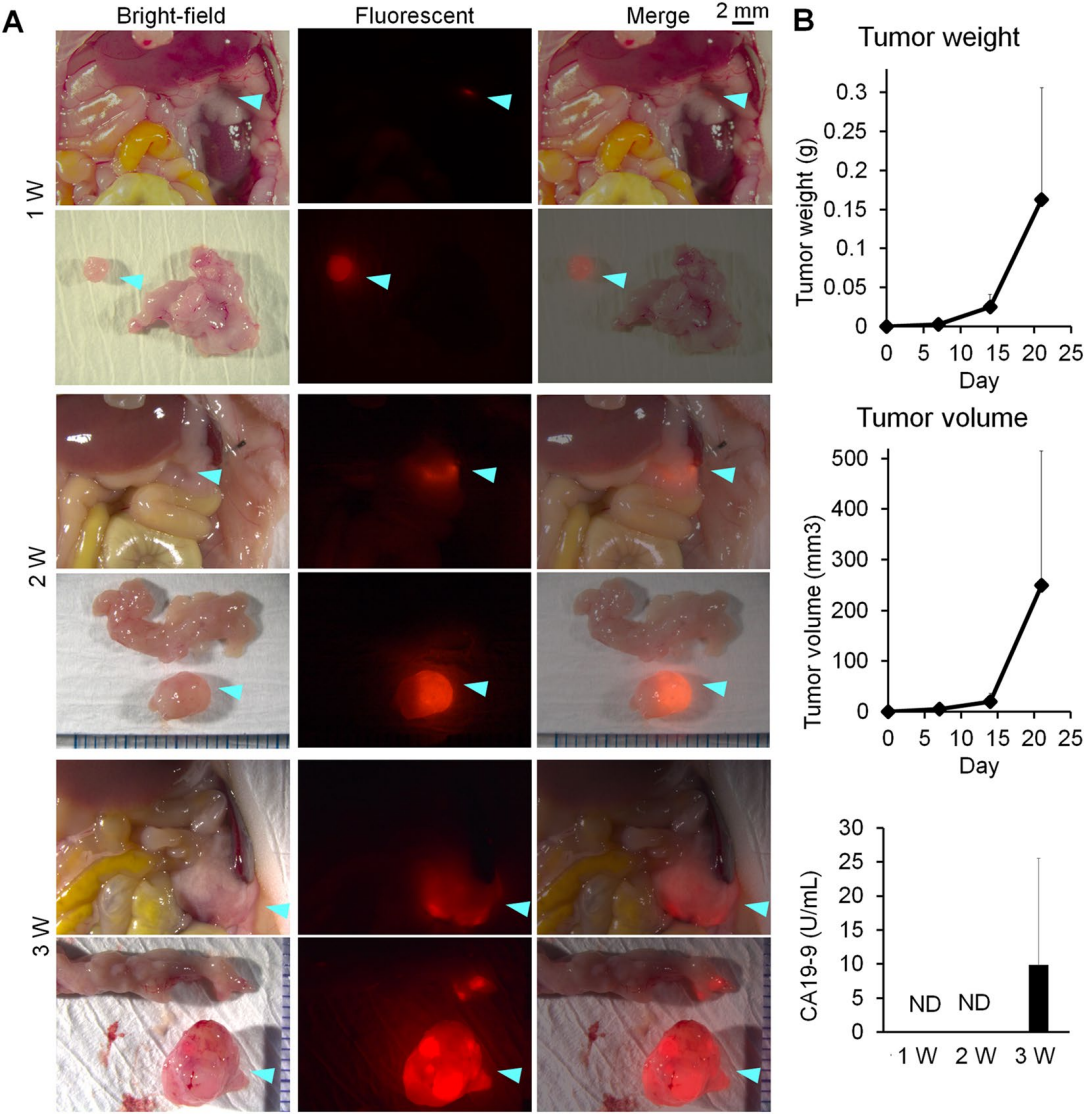
Small resectable orthotopic xPA-1-DC xenograft mouse model. (**A**) Representative stereoscopic fluorescence microscope images for xPA-1-DC xenografts located within the pancreas at 1, 2, and 3 weeks after tumor implantation. Tumors are shown by blue arrowheads. Wide view and isolated tumor within the pancreas are shown in the upper and lower rows in each condition, respectively. Bright-field, red fluorescence, and merged views are shown in the left, middle, and right columns, respectively. (**B**) Tumor weight, tumor volume, and levels of the plasma biomarker CA19-9 in the model at 1, 2, and 3 weeks after tumor implantation (n = 5). ND = not detected.

We successfully established an orthotopic xPA-1-DC xenograft mouse model harboring small resectable PC tumors <1 cm (Fig. 1). One week after tumor implantation, four of the five implanted mice formed tiny tumors within the pancreas (about 2 mm in size), but the other mice did not show obvious tumor mass at this point. At 2 weeks, a small but apparent tumor mass was observed within the pancreas (about 3–5 mm) in all five mice. At 3 weeks, the tumors reached about 5–14 mm in the five examined mice. As shown in Fig. 1B, the tumors in the pancreas showed gradual growth with time, which were relatively uniform in size at 2 weeks after tumor implantation. Moreover, plasma levels of CA19-9 were not detectable at 2 weeks but were detectable at 3 weeks after tumor implantation (Fig. 1B). The levels of CEA were also not detected for up to 3 weeks. Therefore, we determined that 2 weeks post-tumor implantation was an appropriate time to establish this mouse model, which uniformly formed small resectable PC tumors <1 cm (medium dimension of major axis = 4 mm) that would be difficult to detect with conventional plasma biomarkers.

### Feasibility of Immuno-OpenPET imaging for early diagnosis of PC

**Figure 2:**
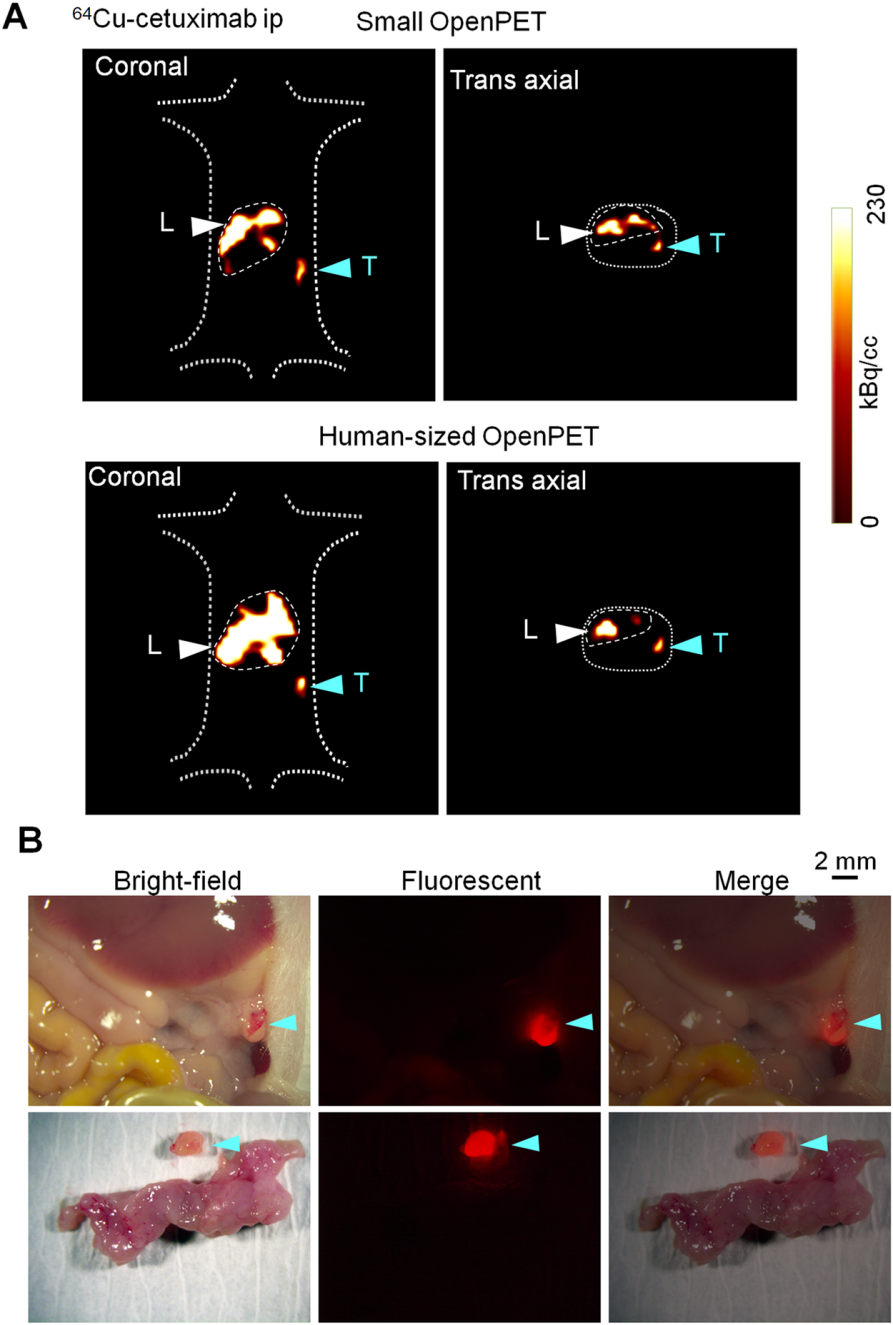
OpenPET imaging with intraperitoneal (ip)-administered ^64^Cu-PCTA-cetuximab. Representative images in the small resectable orthotopic xPA-1-DC xenograft mouse model (2 weeks after tumor implantation) with 3-mm-sized PC tumors. (**A**) OpenPET images (coronal and trans-axial views) obtained with the small OpenPET system (upper) and human-sized OpenPET system (lower). (**B**) Observations of a stereoscopic fluorescence microscope (bright-field, red fluorescence, and merged views). The wide view and isolated tumors with the pancreas are shown in the upper and lower rows, respectively. Tumors are shown by blue arrowheads. L = liver, T = tumor.

**Figure 3:**
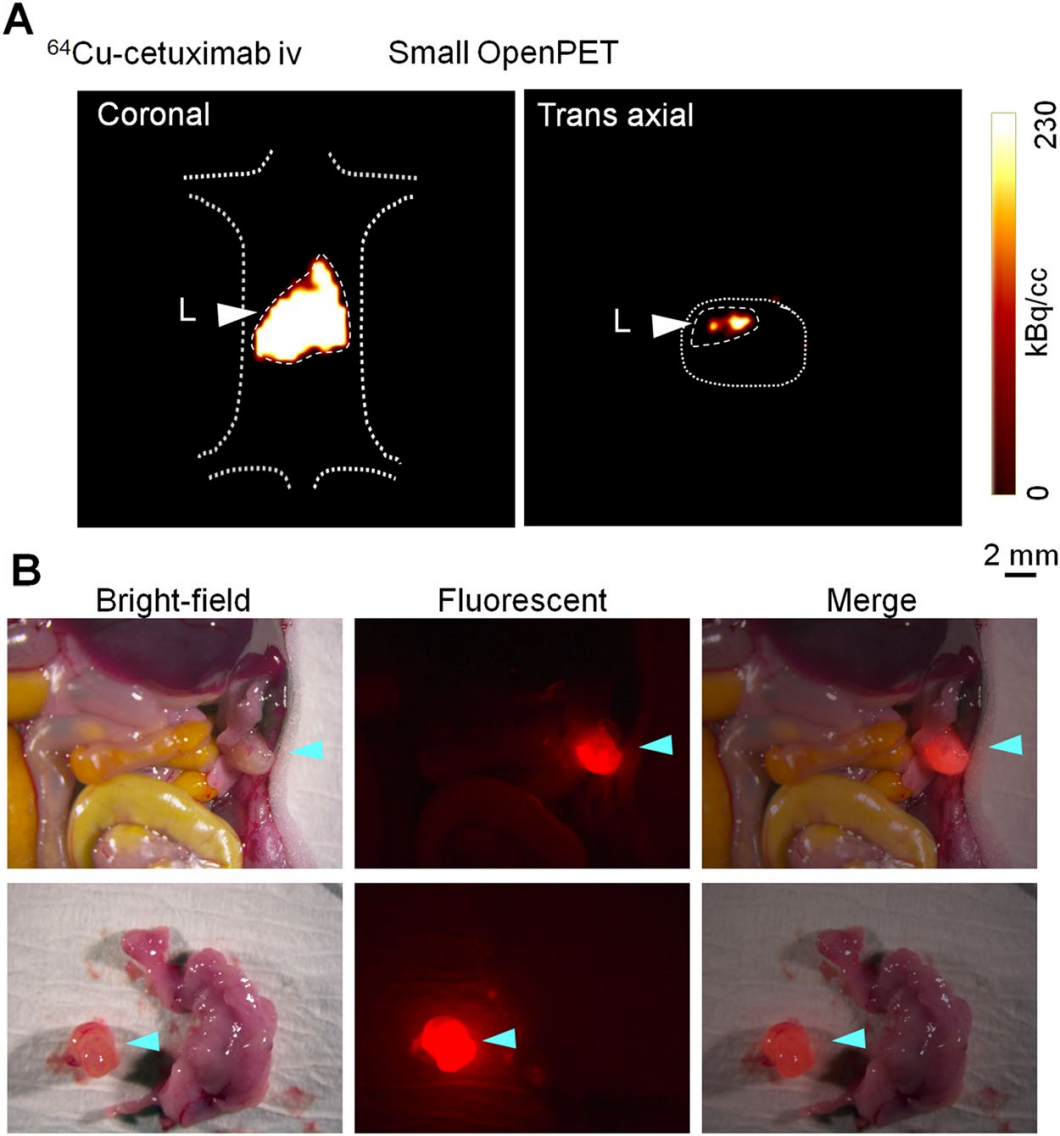
OpenPET imaging with intravenous (iv)-administered ^64^Cu-PCTA-cetuximab. Representative images in the small resectable orthotopic xPA-1-DC xenograft mouse model (2 weeks after tumor implantation). (**A**) OpenPET images obtained with the small OpenPET system (coronal and trans-axial views) and (**B**) observations of a stereoscopic fluorescence microscope (bright-field, red fluorescence, and merged views). For fluorescence microscope observations, the wide view and isolated tumor with pancreas are shown in the upper and lower rows, respectively. Tumors are shown by blue arrowheads. L = liver.

**Figure 4:**
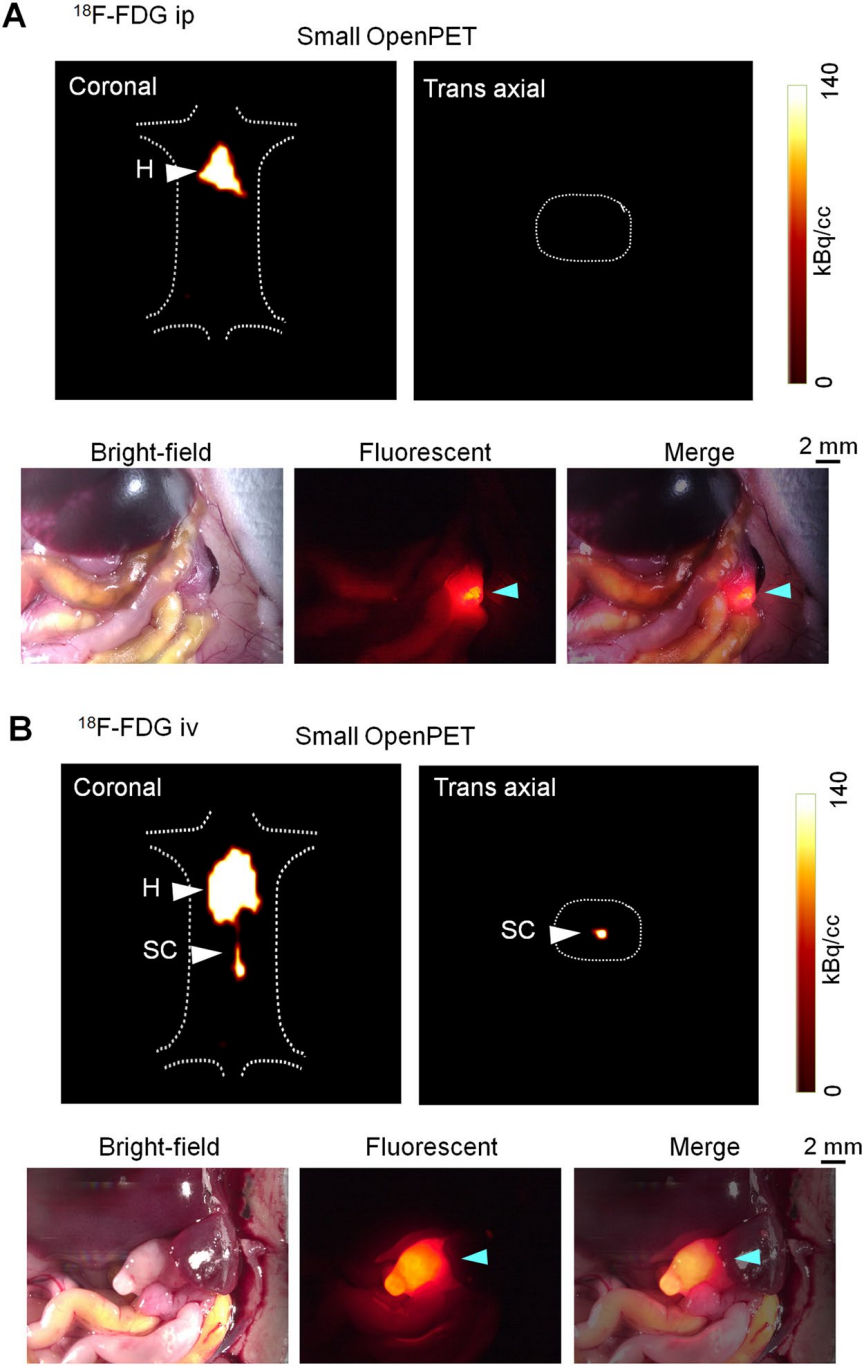
OpenPET imaging with intraperitoneal (ip) or intravenous (iv)-administered ^18^F-FDG. Representative images in the small resectable orthotopic xPA-1-DC xenograft mouse model (2 weeks after tumor implantation) with ip- or iv-administered ^18^F-FDG (**A** and **B**, respectively). OpenPET images obtained with the small OpenPET system (coronal and trans-axial views) and observations of a stereoscopic fluorescence microscope (bright-field, red fluorescence, and merged views) are shown in upper and lower rows for each condition, respectively. Tumors are shown by blue arrowheads. H = heart, SC = spinal cord.

Immuno-OpenPET imaging with intraperitoneal-administered ^64^Cu-PCTA-cetuximab clearly detected small resectable PC tumors (<1 cm), and the tumor location was confirmed to be consistent between OpenPET imaging and fluorescent observation after laparotomy in all examined mice (Table 1, Fig. 2, Supplementary Fig. S3). No obvious distant metastasis was detected in this model. Immuno-OpenPET with both the small and human-sized OpenPET systems clearly detected PC lesions ≥3 mm in this model. OpenPET imaging with intravenous-administered ^64^Cu-PCTA-cetuximab could not detect PC in this model, despite observation of these small tumors with fluorescence microscopy after laparotomy in all examined mice (Table 1, Fig. 3). No apparent signals were detected in the normal organs of the peritoneal cavity except for the liver in both the intraperitoneal and intravenous-administered ^64^Cu-PCTA-cetuximab xenograft models (Figs. 2 and 3) and in sham operation mice (Supplementary Fig. S4). In either ^18^F-FDG group, the OpenPET imaging could not detect the PCs (Table 1, Fig. 4). For the ^18^F-FDG imaging, the heart and/or spinal cord was detectable in both xenograft models (Fig. 4) and sham operation mice (Supplementary Fig. S5). This distribution pattern of ^18^F-FDG (administered intraperitoneally or intravenously) in mice was consistent with that reported previously^28,29^.

**Table 1:** Summary of OpenPET imaging with ^64^Cu-PCTA-cetuximab and ^18^F-FDG (ip or iv).

Mouse*	Tumor dimension (mm)		PET^†^
	Major axis	Minor axis	
^**64**^**Cu-PCTA-cetuximab ip**			
Mouse-1-1	3	2	Positive^§^
Mouse-1-2	3	2	Positive^§^
Mouse-1-3	4	3	Positive^§^
Mouse-1-4	4	3	Positive^§^
Mouse-1-5	3	3	Positive^§^
Mouse-1-6	9	5	Positive^§^
^**64**^**Cu-PCTA-cetuximab iv**			
Mouse-2-1	8	4	Negative
Mouse-2-2	3	3	Negative
Mouse-2-3	5	3	Negative
Mouse-2-4	7	7	Negative
Mouse-2-5	4	3	Negative
Mouse-2-6	4	3	Negative
^**18**^**F-FDG ip**			
Mouse-3-1	4	3	Negative
Mouse-3-2	8	4	Negative
Mouse-3-3	4	4	Negative
Mouse-3-4	7	4	Negative
^**18**^**F-FDG iv**			
Mouse-4-1	4	4	Negative
Mouse-4-2	5	3	Negative
Mouse-4-3	4	4	Negative
Mouse-4-4	8	4	Negative

*The OpenPET images of the following mice are shown in various figures: mouse-1-1, Fig. 2; mouse-1-3, Supplementary Fig. S3; mouse-2-2, Fig. 3; mouse-3-3, Fig. 4A; mouse-4-2, Fig. 4B. ^†^Imaging with the small OpenPET system. Positive and negative indicate detection and no detection of tumor, respectively, by OpenPET imaging in the mice. ^§^Similar detection with the human-sized Open-PET system.

**Figure 5:**
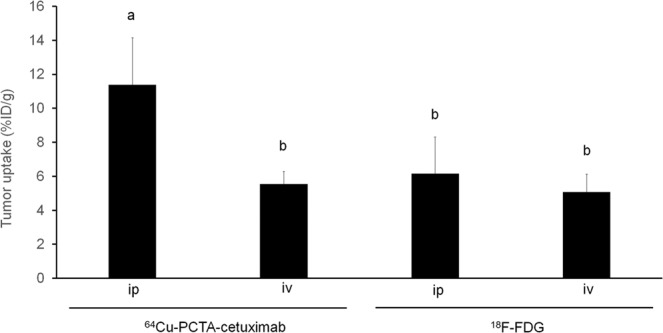
Tumor uptake of intraperitoneal (ip) or intravenous (iv)-administered ^64^Cu-PCTA-cetuximab and ^18^F-FDG in xPA-1-DC xenografts. Values are shown as the mean ± SD. Means marked by different letters (a and b) are significantly different (*P* < 0.05).

Tumor uptake (%ID/g) of intraperitoneal-administered ^64^Cu-PCTA-cetuximab was significantly higher than that of intravenous-administered ^64^Cu-PCTA-cetuximab and ^18^F-FDG regardless of the administration route (Fig. 5). Table 2 (also see Supplementary Figs. S6 and S7) summarizes the uptake ratio of the tumor to that of adjacent organs (small intestine, large intestine, spleen, pancreas, and stomach), showing that intraperitoneal-administered ^64^Cu-cetuximab showed the highest values overall. OpenPET imaging did not detect tumors around 2 mm in the small resectable PC orthotopic xPA-1-DC xenograft mouse model at 1 week after tumor implantation (n = 5), indicating that 2-mm-sized tumors were too small to be detected with this system (Supplementary Fig. S8).

**Table 2:** Uptake ratio of xPA-1-DC tumors to adjacent organs in ^64^Cu-PCTA-cetuximab and ^18^F-FDG (ip or iv).

	Uptake (%ID/g)*	Ratio (Tumor/Organ uptake)
^**64**^**Cu-PCTA-cetuximab ip**		
Small intestine	1.85 ± 1.15	6.16
Large intestine	2.31 ± 1.45	4.93
Spleen	3.71 ± 2.60	3.07
Pancreas	2.71 ± 2.06	4.20
Stomach	1.26 ± 1.01	9.04
Tumor	11.39 ± 2.74	—
^**64**^**Cu-PCTA-cetuximab iv**		
Small intestine	2.50 ± 0.15	2.22
Large intestine	2.91 ± 0.45	1.90
Spleen	4.02 ± 0.29	1.38
Pancreas	1.64 ± 0.07	3.38
Stomach	2.18 ± 0.36	2.54
Tumor	5.54 ± 0.73	—
^**18**^**F-FDG ip**		
Small intestine	2.83 ± 0.13	2.18
Large intestine	2.63 ± 0.63	2.34
Spleen	3.02 ± 0.21	2.04
Pancreas	3.53 ± 0.71	1.75
Stomach	2.04 ± 0.41	3.02
Tumor	6.16 ± 2.15	—
^**18**^**F-FDG iv**		
Small intestine	1.81 ± 0.21	2.81
Large intestine	2.04 ± 0.47	2.49
Spleen	2.36 ± 0.42	2.15
Pancreas	3.38 ± 2.55	1.50
Stomach	2.40 ± 0.54	2.12
Tumor	5.08 ± 1.05	—

*For ^64^Cu-PCTA-cetuximab (ip or iv), the values were referred from^18^. For ^18^F-FDG (ip or iv), the values were referred from Supplementary Figs. S6 and S7.

### Efficacy of immuno-OpenPET-guided surgery

During surgical resection, intraoperative OpenPET clearly identified the localization of PC lesions ≥3 mm within the pancreas, which were not visible with the naked eye. Thus, OpenPET was helpful to resect the tumors and confirm the absence of residual tumor signals during the operation, and the presence of fluorescence derived from xPA-1-DC tumors was confirmed in the resected tissues after surgery (Video S1). An approximately 10–30-s data acquisition was sufficient to identify tumors during surgical procedures. There were no procedural or anesthesia-related fatalities after immuno-OpenPET-guided surgery.

**Figure 6:**
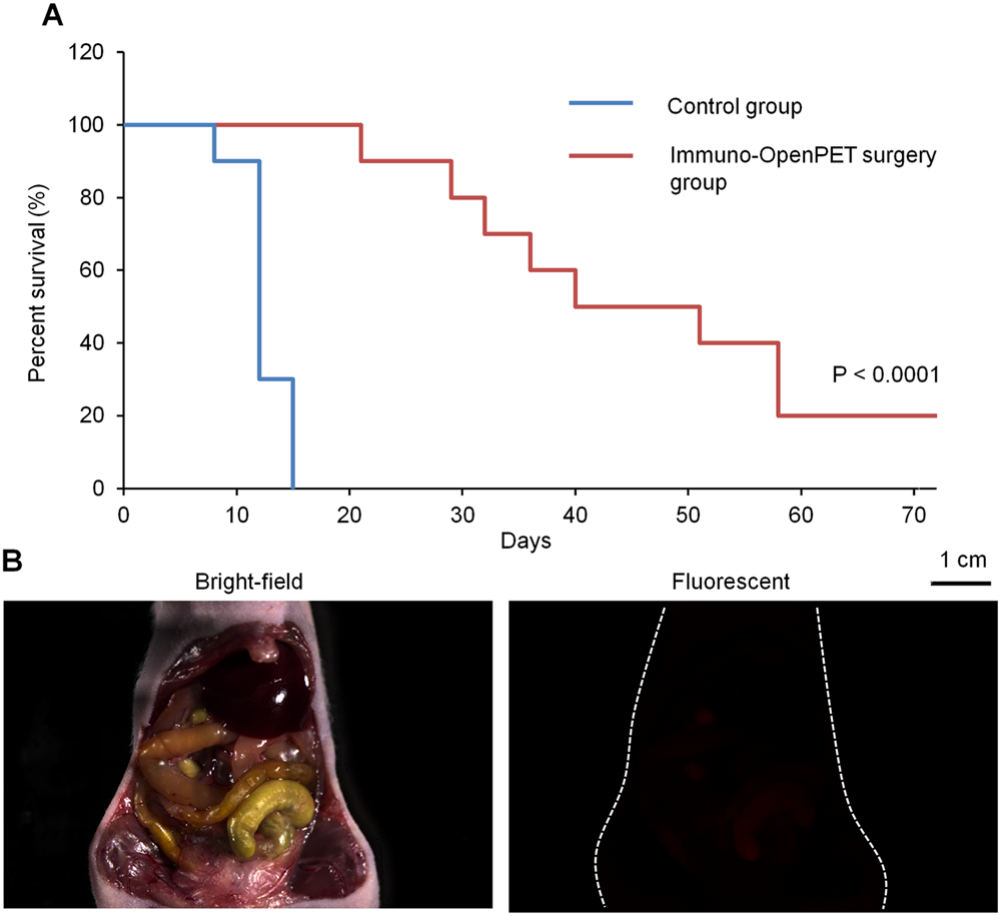
Immuno-OpenPET-guided surgery. (**A**) Survival curves for immuno-OpenPET surgery with intraperitoneal (ip)-administered ^64^Cu-PCTA-cetuximab (immuno-OpenPET surgery) and non-treated control groups (*n* = 10/group) in the small resectable orthotopic xPA-1-DC xenograft mouse model (2 weeks after tumor implantation). There was a significant difference in survival between the immuno-OpenPET surgery group and control group (*P* < 0.0001). (**B**) Representative images of stereoscopic fluorescence microscope images (bright-field and red fluorescence views) at day 72 in a mouse that underwent immuno-OpenPET surgery with ^64^Cu-PCTA-cetuximab. There were no detectable tumor lesions.

Survival was significantly extended in the immuno-OpenPET surgery groups compared with that in the control group, with 3.8-fold greater median survival (45.5 and 12 days, respectively; *P* < 0.0001; Fig. 6A). All mice in the control group were dead by day 15, while mice in the immuno-OpenPET surgery group showed 100% survival at day 15 and 20% survival at day 72 (2/10 mice). In the two surviving mice of the immuno-OpenPET surgery group, there were no detectable tumor lesions at day 72 (Fig. 6B). No mice in any group showed a decrease in body weight over 20% relative to the initial body weight up to the experimental endpoint. Mice in the control group died of growth of primary PC, and 8 of 10 mice in the immuno-OpenPET surgery groups died of local recurrence and peritoneal metastasis. In tumor-free mice that underwent similar surgery, the general condition and body weight were not adversely affected (Supplementary Fig. S9).

## Discussion

Using a small resectable human PC orthotopic xenograft mouse model, we demonstrated that immuno-OpenPET with intraperitoneal-administered ^64^Cu-PCTA-cetuximab was useful for not only early diagnosis but also image-guided surgery of PC. The immuno-OpenPET system allowed for early diagnosis and accurate resection for PC tumors ≥3 mm. Considering that the surgical resection of PC < 1 cm was shown to increase survival in clinical studies^6–8^ and that detection of small PC < 1 cm is difficult using current imaging methods, immuno-OpenPET is a promising solution to improve the prognosis of patients with PC. Thus, the application of immuno-OpenPET might be a useful option for suspected PC cases that are screened by plasma biomarkers but diagnosed as negative or ambiguous with current imaging modalities. If a positive diagnosis of PC is obtained with immuno-OpenPET, immuno-OpenPET-guided surgery could be a suitable choice for achieving a better outcome.

Moreover, intraperitoneal-administered ^64^Cu-PCTA-cetuximab resulted in higher tumor uptake and uptake ratios of the tumor to the adjacent organs than intravenous-administered ^64^Cu-PCTA-cetuximab and intraperitoneal/intravenous-administered ^18^F-FDG. This indicates that the combination of ^64^Cu-PCTA-cetuximab and intraperitoneal administration is important to achieve better detection of small PC by the OpenPET system. Thus, the specialized skill required for intraperitoneal administration justifies the cost for the clinical development of this method given its benefits. Nevertheless, immuno-OpenPET could not detect PC tumors of around 2 mm, which is a limitation of the resolution of this system (Supplementary Table S1); hence, follow-up investigations should be considered to avoid the potential underdiagnosis of PC smaller than this limitation for its future clinical use. Furthermore, to apply this technology to clinical practice, the independent processes for development of the new PET system and radiopharmaceutical are required in future.

The main advantage of OpenPET over conventional PET systems or other developed high-resolution PET systems^30^ is that this system can achieve not only early diagnosis but also facilitate image-guided therapies. Indeed, immuno-OpenPET could detect and accordingly help to resect small PC tumors within the pancreas that were invisible to the naked eye during surgery, and the immuno-OpenPET-guided surgery significantly prolonged survival. This suggests the benefit of this technology for PC prognosis. Although we observed a clear survival benefit from the immuno-OpenPET-guided surgery, 80% of the treated mice ultimately experienced local recurrence and peritoneal metastasis in this model. To avoid these recurrences, adjuvant chemotherapy or adjuvant therapy with intraperitoneal-administered ^64^Cu-PCTA-cetuximab^19^ should be considered as additional options.

For the future clinical application of image-guided surgery with immuno-OpenPET, robotic surgery, which is already used for PC surgery in clinical practice^31^, should be considered to avoid radiation exposure for the surgeons (Supplementary Fig. S10). We previously reported that human-sized OpenPET can be used under carbon ion therapy^32^. Additionally, in a recent multi-institutional clinical study, carbon ion therapy showed favorable outcomes with limited toxicities for PC treatment^33^. Based on these findings, it is worthwhile to pursue the combination of immuno-OpenPET with carbon ion therapy as a future potential non-invasive therapeutic option for small resectable PC.

This study has several limitations. At the time of PC development of <1 cm, the traditional plasma biomarkers CA19-9 and CEA were not detected in the small resectable xenograft mouse model; however, we did not investigate the ability of new plasma biomarkers that are currently under development to potentially detect PC at this early stage. Thus, the efficacy of the combination of these new plasma biomarkers and immuno-OpenPET detection will be examined in future clinical studies. We also demonstrated that immuno-OpenPET-guided surgery can detect small PC and prolonged survival compared to that of non-treated mice. However, the benefit of immuno-OpenPET diagnosis over conventional surgical procedures remains unclear; thus, further clinical studies are necessary to investigate the complete benefits of this option. Furthermore, the optimal dose, detectability, biodistribution, dosimetry, and safety of intraperitoneal-administered ^64^Cu-cetuximab with immuno-OpenPET in humans should be confirmed in future clinical studies.

## Conclusion

Immuno-OpenPET with ^64^Cu-PCTA-cetuximab was useful for the early diagnosis and image-guided surgery of PC using a clinically relevant small resectable xenograft mouse model. The immuno-OpenPET imaging could detect PC tumors ≥3 mm and the image-guided surgery allowed for accurate resection and prolonged the survival of mice. Our findings suggest that immuno-OpenPET offers a novel approach for the early diagnosis and treatment of PC, and its combined use with currently developing plasma biomarkers is warranted in the near future.

## Supplementary information

Supplementary Video S1. Immuno-OpenPET-guided surgery for small resectable PC.

Supplementary Information.

## Data Availability

All data generated or analyzed during this study are included in this published article (and its Supplementary Information files).
