# Supplementary material for: Immuno-OpenPET: a novel approach for early diagnosis and image-guided surgery for small resectable pancreatic cancer
Source: Sci Rep. 2020 Mar 10;10:4143. doi: 10.1038/s41598-020-61056-5 (PMC7064510; doi:10.1038/s41598-020-61056-5)
Supplement: Supplementary file 2 — Supplementary Information. [file 41598_2020_61056_MOESM2_ESM.docx]

**Supplementary Information**

**Immuno-OpenPET: a novel approach for early diagnosis and image-guided surgery for small resectable pancreatic cancer**

Yukie Yoshii^1^, Hideaki Tashima^1^, Yuma Iwao^1^, Eiji Yoshida^1^, Hidekatsu Wakizaka^1^, Go Akamatsu^1^, Taiga Yamaya^1^, Hiroki Matsumoto^2^, Mitsuyoshi Yoshimoto^3^, Chika Igarashi^1^, Fukiko Hihara^1^, Tomoko Tachibana^1^, Ming-Rong Zhang^1^, Kotaro Nagatsu^1^, Aya Sugyo^1^, Atsushi B. Tsuji^1^, Tatsuya Higashi^1^

*^1^National Institute of Radiological Sciences, National Institutes for Quantum and Radiological Science and Technology, Chiba 263-8555, Japan; ^2^Nihon Medi-Physics Co., Ltd., Tokyo 136-0075, Japan; ^3^Division of Functional Imaging, National Cancer Center Hospital East, Kashiwa, Chiba 277-8577, Japan*

**Supplementary Table S1.** Summary of technical features of small and human-sized OpenPET systems.

| Features | Small OpenPET | Human-sized OpenPET |
| --- | --- | --- |
| Scintillation crystal materials | Gd_2_SiO_5_ (GSO) | Zr-doped GSO |
| Photodetector | 64-ch flat panel position sensitivity photomultiplier tube (PS-PMT) | Super bialkalii 64-ch flat panel PS-PMT |
| Size of crystal element | 2.8 × 2.8 × 7.5 mm^3^ | 2.8 × 2.8 × 7.5 mm^3^ |
| Number of crystals per detector | 16 × 16 × 4 layers | 16 × 16 × 4 layers |
| Number of detector blocks | 16 | 160 |
| Number of detector rings | 2 | 4 |
| Axial field of view | 102 mm | 215 mm |
| Ring diameter | 250 mm | 660 mm |
| Spatial resolution | 2.6 ± 0.2 mm | 2.1 ± 0.4 mm |

See details in references[^1-4^](#_ENREF_1).

**
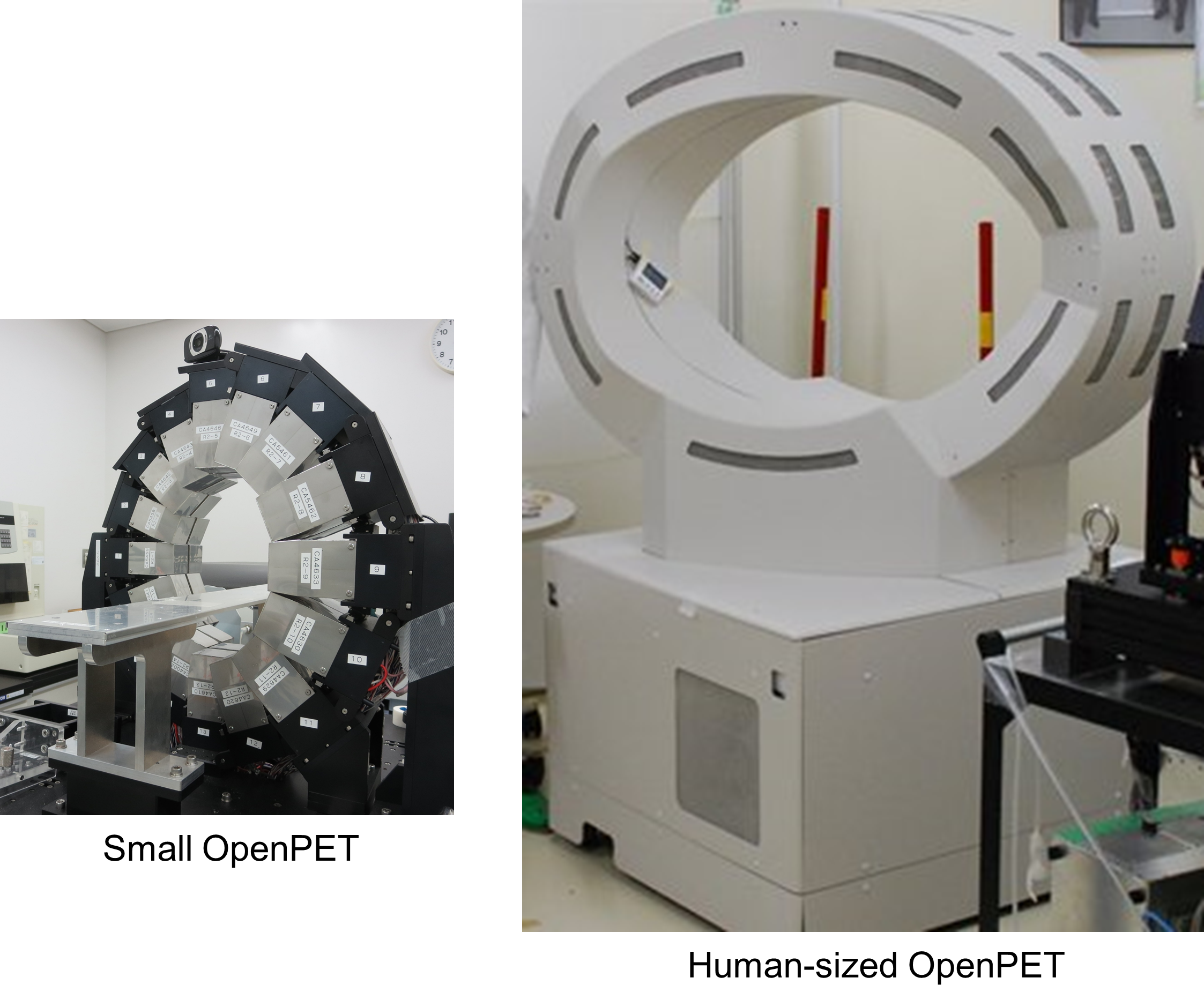
**

**Supplementary Figure S1. OpenPET systems.** Photographs of the small OpenPET system (left) and human-sized OpenPET system (right). See details in references[^1-4^](#_ENREF_1).


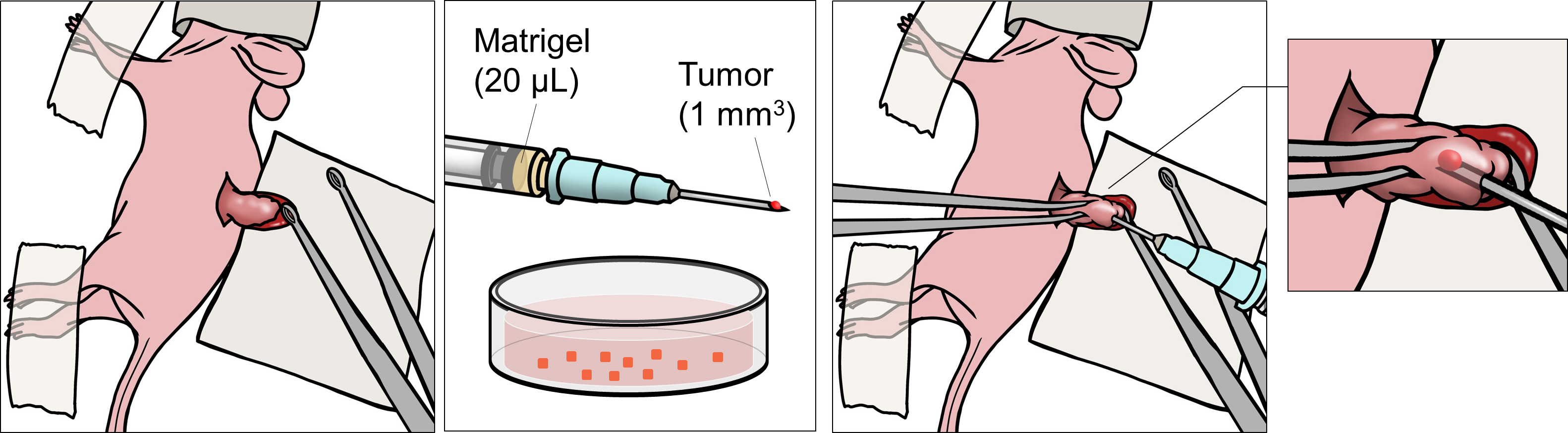


**Supplementary Figure S2. Schematic of the establishment of the** **small resectable orthotopic xPA-1-DC** **xenograft mouse model.** Procedures for implantation of a piece of xPA-1-DC tumor with a syringe to establish the mouse model. The images were illustrated by Sayaka Hanadate.

**
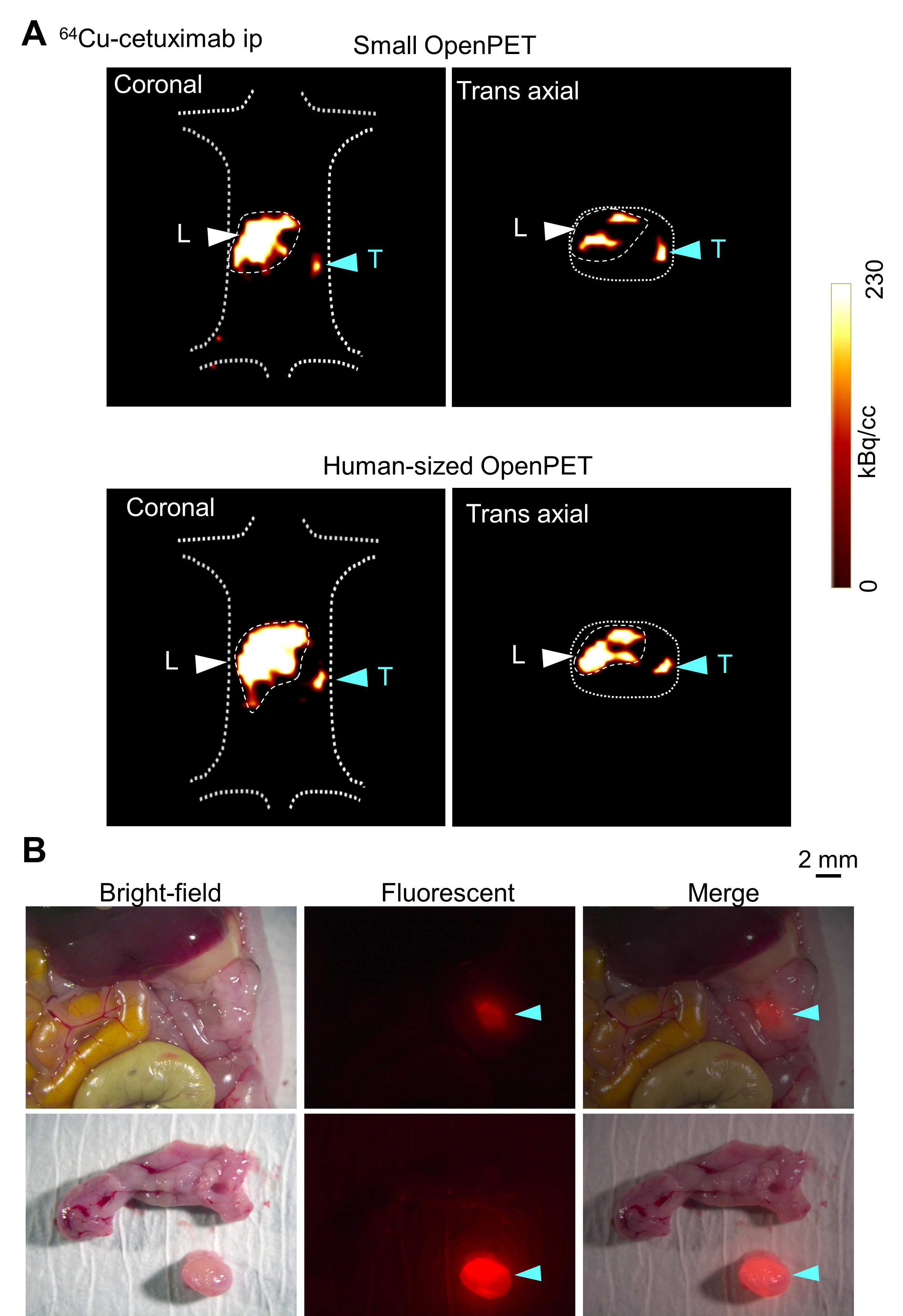
**

**Supplementary Figure S3. Model mouse with a 4-mm-sized tumor for OpenPET imaging with intraperitoneal (ip)-administered ^64^Cu-PCTA-cetuximab**. Small resectable orthotopic xPA-1-DC xenograft mouse model (2 weeks after tumor implantation) with a 4-mm-sized tumor. (A) OpenPET images (coronal and trans axial views) obtained with the small OpenPET system (upper) and human-sized OpenPET system (lower). (B) Stereoscopic fluorescence microscope images (bright-field, red fluorescence, and merged views). The wide view and isolated tumor with the pancreas are shown in upper and lower rows, respectively. Tumors are shown by blue arrowheads. L = liver, T = tumor.


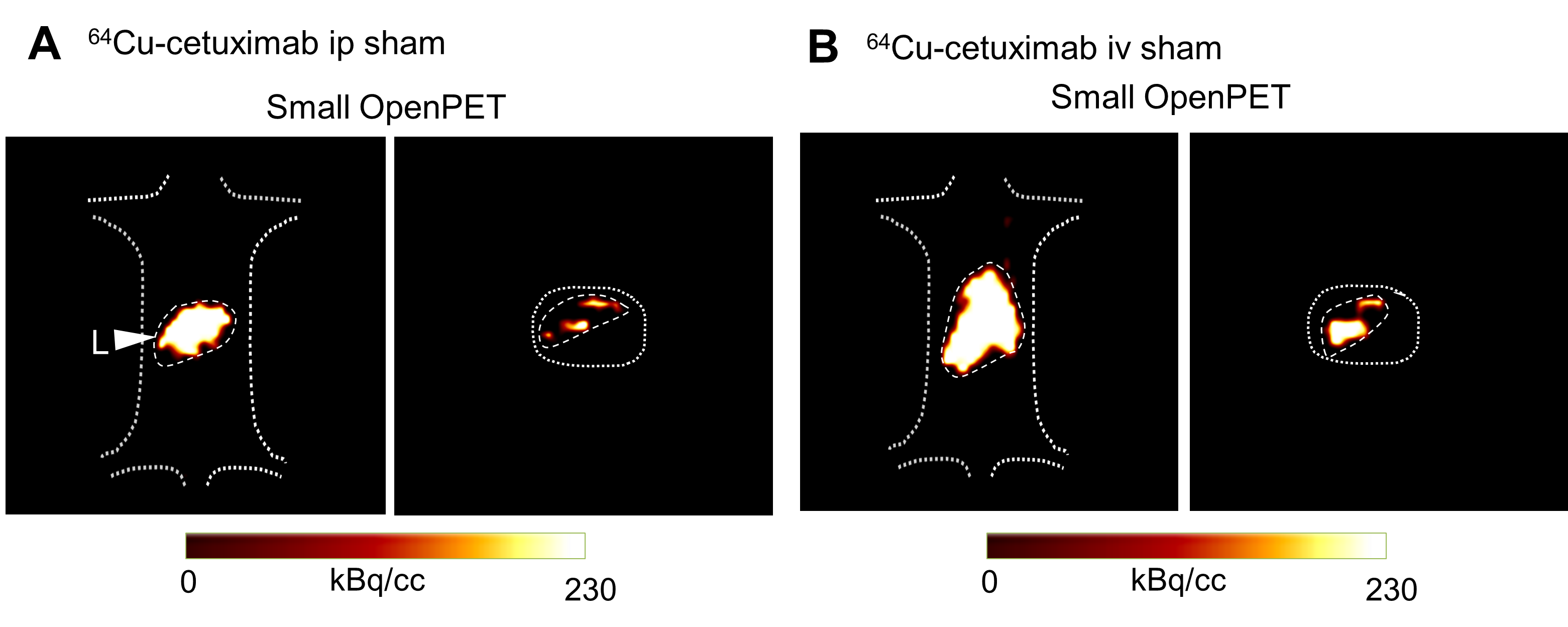


**Supplementary Figure S4.** **OpenPET imaging with tumor-free sham operation mice intraperitoneal (ip) or intravenous (iv)-administered ^64^Cu-PCTA-cetuximab.** Mice with sham operation without tumor implantation were treated in the similar manner as the early pancreatic cancer orthotopic xPA-1-DC xenograft mouse model (2 weeks after tumor implantation). The OpenPET imaging was performed with the same procedures represented in Figure 3 and Figure 4, respectively. L = liver.

**
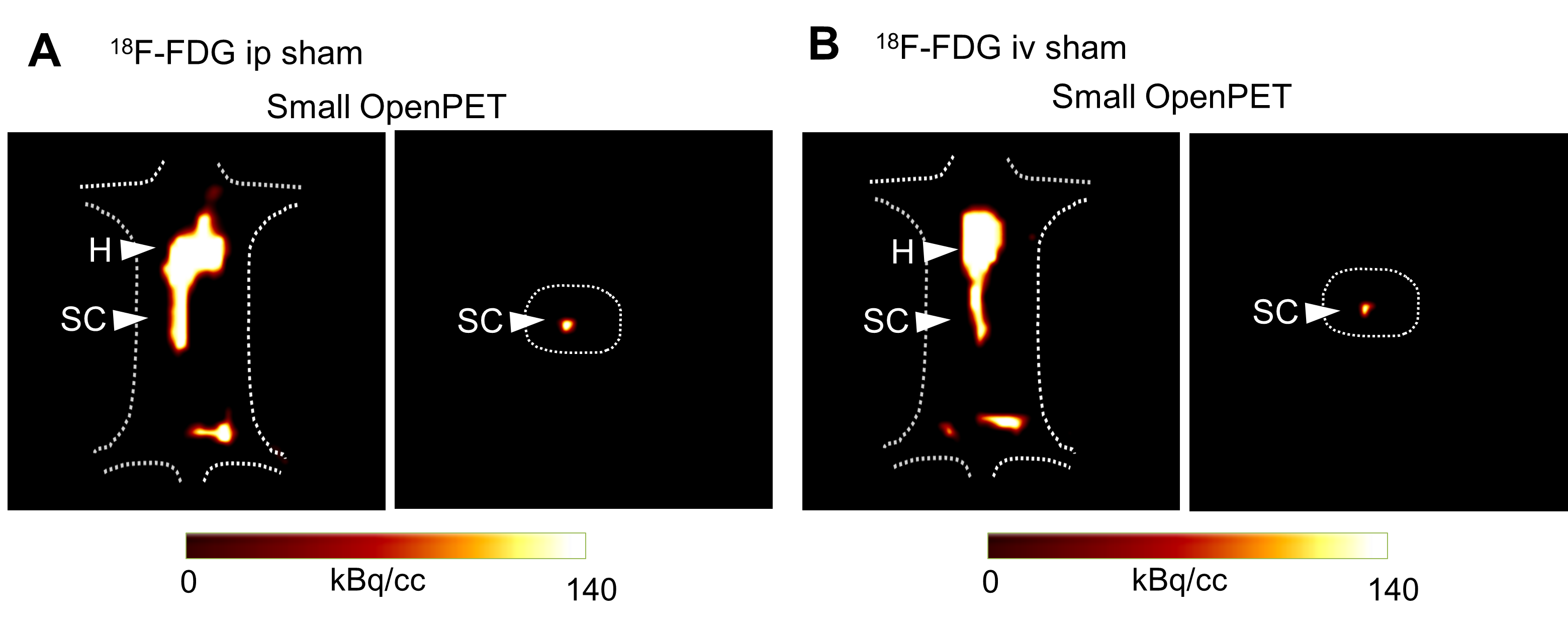
**

**Supplementary Figure S5. OpenPET imaging with tumor-free sham operation mice intraperitoneal (ip) or intravenous (iv) administered ^18^F-FDG.** Mice with sham operation without tumor implantation were treated in the similar manner as the early pancreatic cancer orthotopic xPA-1-DC xenograft mouse model (2 weeks after tumor implantation). The OpenPET imaging was performed with the same procedures represented in Figure 5. Signals from the heart and/or spinal cord were detected, which were consistent with previous reports[^5^](#_ENREF_5)^,^[^6^](#_ENREF_6). H = heart, SC = spinal cord.


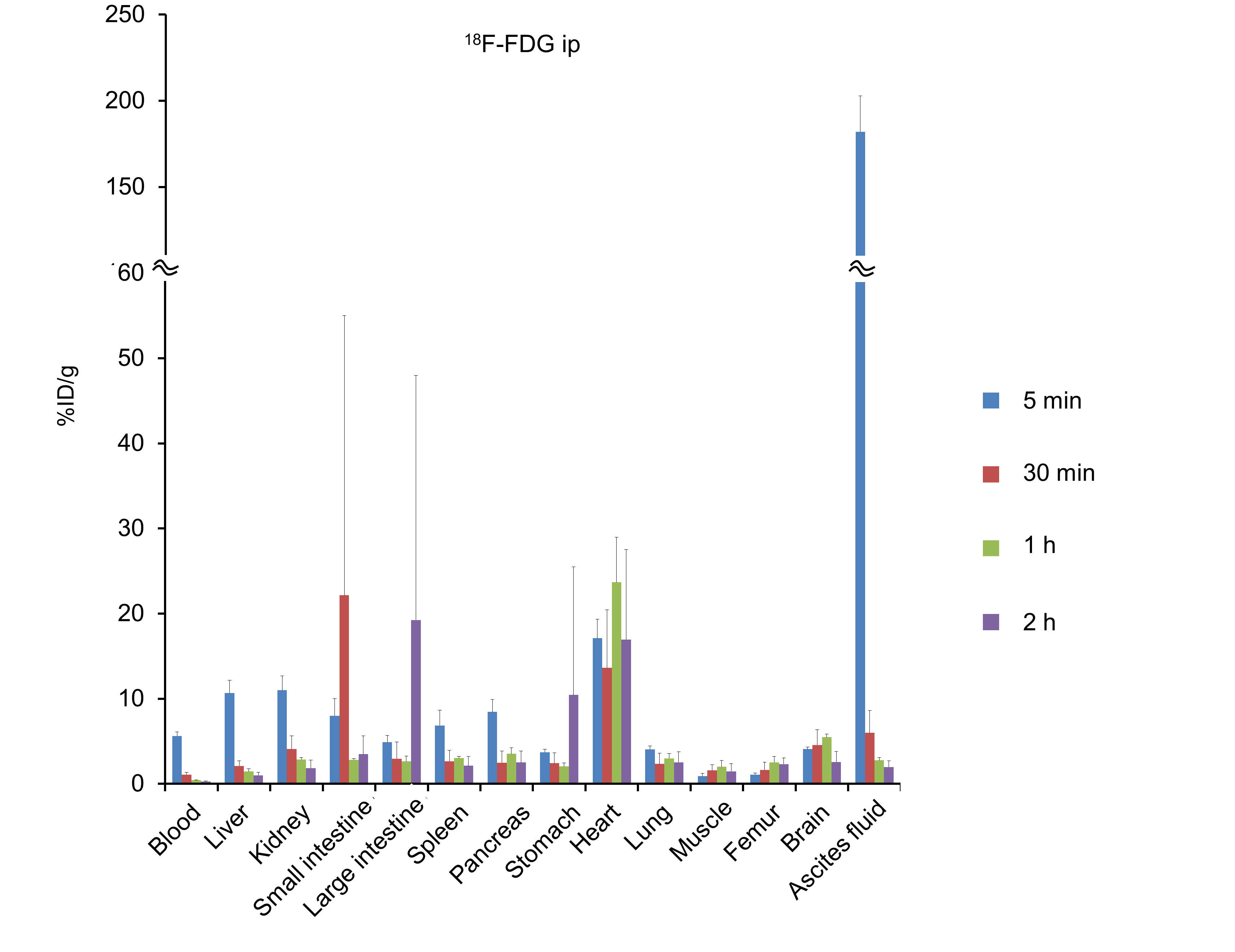


**Supplementary Figure S6. Biodistribution of** **intraperitoneal (ip)-administered ^18^F-FDG.** Distribution of ^18^F-FDG in the organs of interest in mice at 5 min, 30 min, 1 h, and 2 h (n = 4). Values are shown as the mean ± SD. The biodistribution study was performed as previously described[^7^](#_ENREF_7). The biodistribution data were calculated as the %ID/g.

**
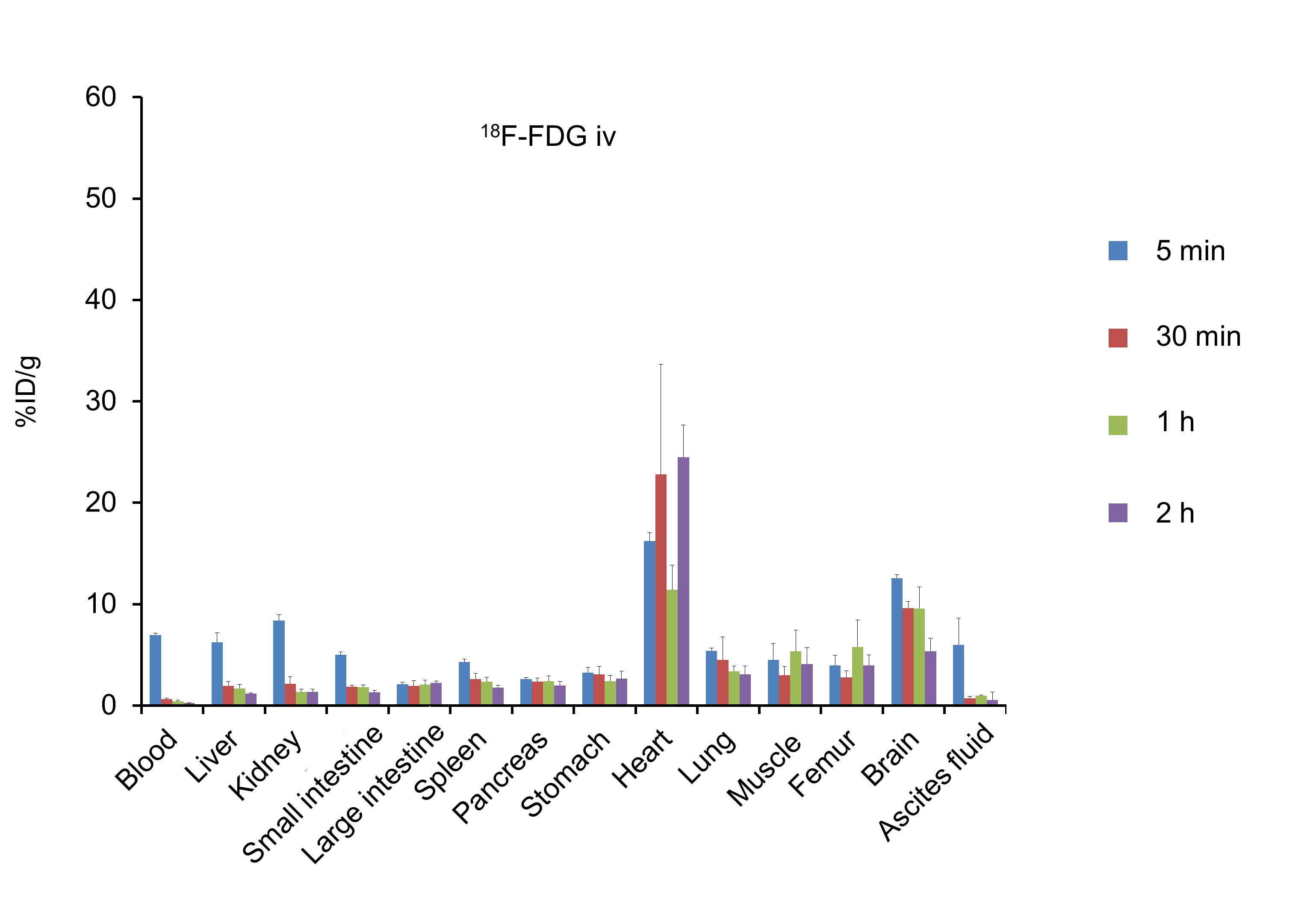
**

**Supplementary Figure S7. Biodistribution of** **intravenous (iv)-administered ^18^F-FDG.** Distribution of ^18^F-FDG in the organs of interest in mice at 5 min, 30 min, 1 h, and 2 h (n = 4). Values are shown as the mean ± SD. The biodistribution study was performed as previously described[^7^](#_ENREF_7). The biodistribution data were calculated as the %ID/g.

**
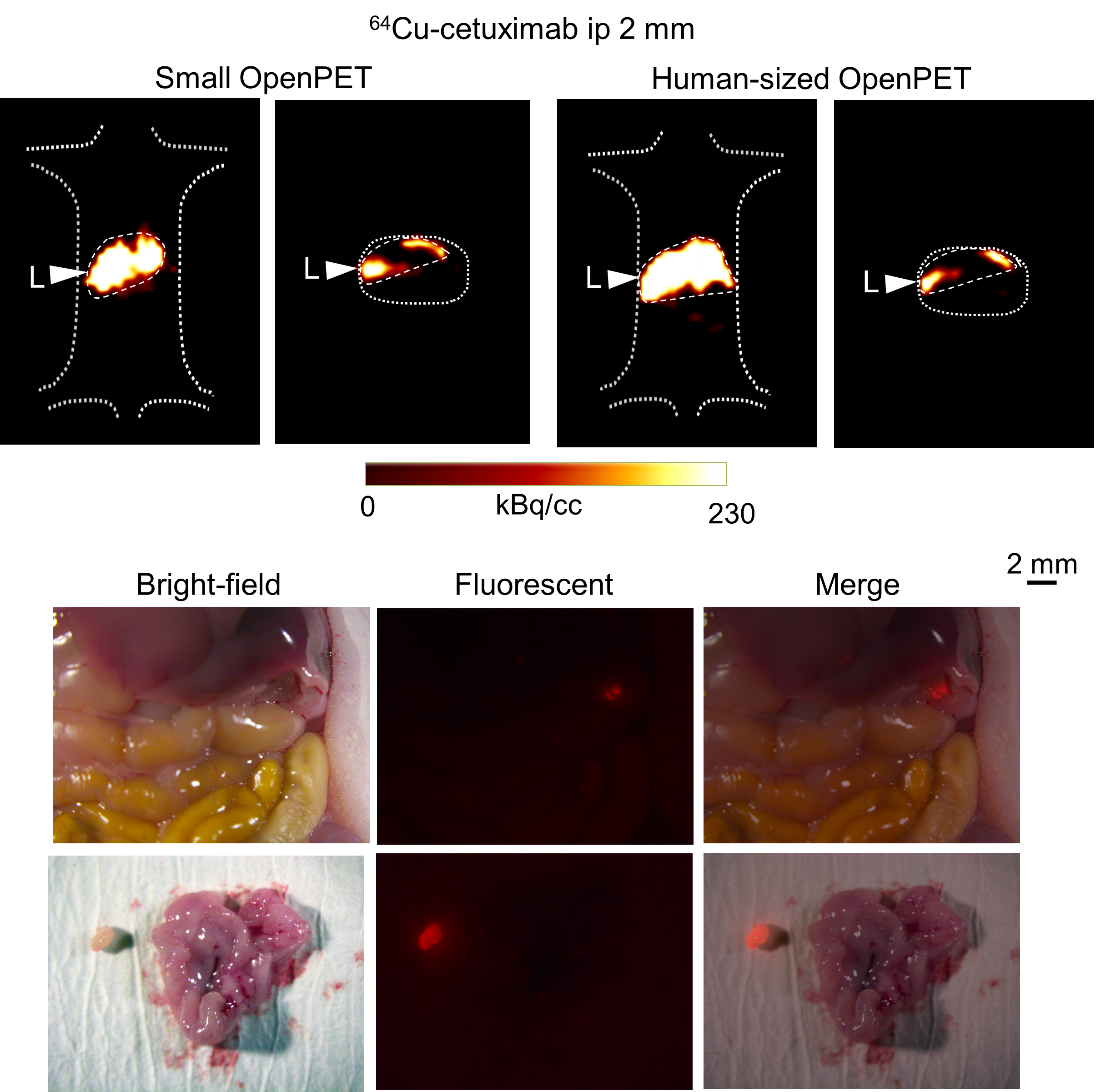
**

**Supplementary Figure S8. OpenPET imaging with intraperitoneal (ip)-administered ^64^Cu-PCTA-cetuximab** **in the small resectable orthotopic xPA-1-DC xenograft mouse model with a 2-mm tumor**. To evaluate the use of the immuno-OpenPET for smaller tumors, OpenPET imaging with the early pancreatic cancer (PC) orthotopic xPA-1-DC xenograft mouse model at 1 weeks after tumor implantation (n = 5) was tested in the similar manner to that at 2 weeks. Representative images are shown. The immuno-OpenPET with ip-administered ^64^Cu-PCTA-cetuximab did not clearly detect 2-mm sized PC tumors. OpenPET images (coronal and trans-axial views) and stereoscopic fluorescence microscope images (bright-field, red fluorescence, and merged views) are shown in the upper and lower rows, respectively. For OpenPET images, images obtained with a small OpenPET system (left) and human-sized OpenPET system (right) are shown. For fluorescence microscope observations, a wide view and isolated tumor with the pancreas are shown in the upper and lower rows, respectively. L = liver.

**
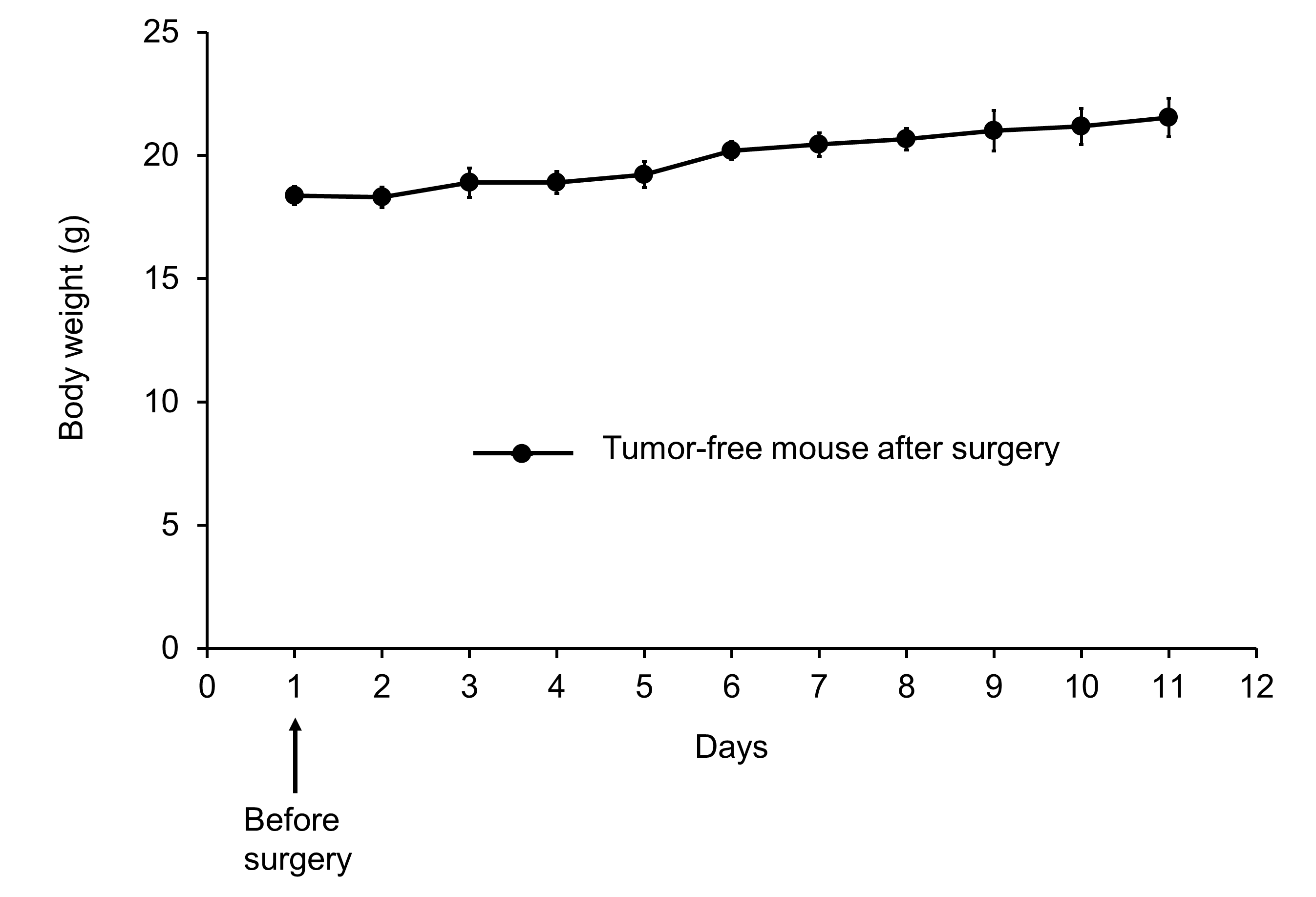
**

**Supplementary Figure S9. Body weight dynamics in tumor-free mice after surgery.** To confirm the absence of adverse effects related to the surgical procedures with partial pancreas resection in the *in vivo* study of OpenPET-guided surgery (Fig. 6), partial pancreas resection surgery was performed in five tumor-free mice in a manner similar to that shown in Fig. 6 (at day 1). The general conditions and body weight were unperturbed in these mice. Values are shown as the mean ± SD in body weight.


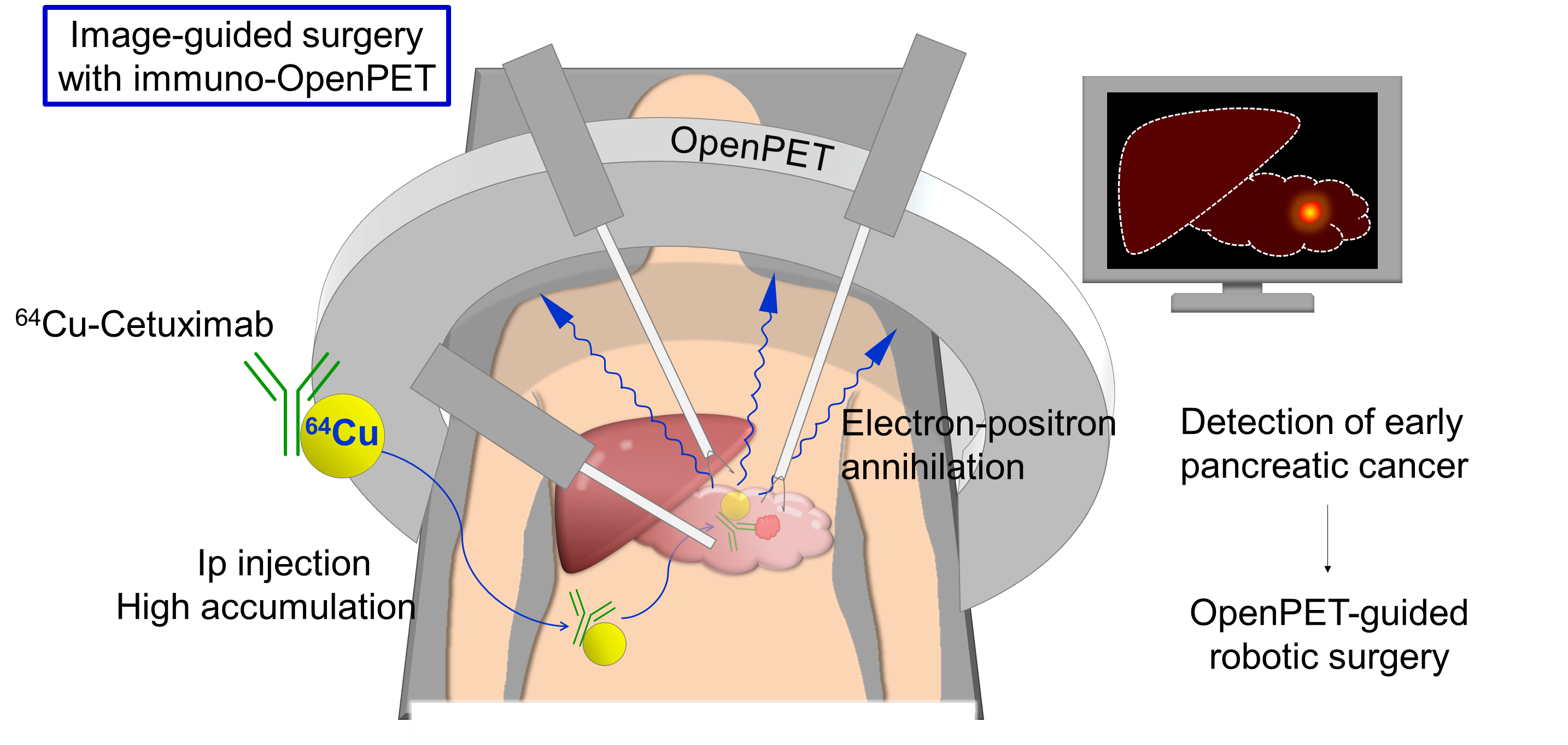


**Supplementary Figure S10. Schematic of immuno-OpenPET with the combination of robotic surgery.** Immuno-OpenPET would be useful with the combination with robotic surgery. The images were illustrated by Sayaka Hanadate.

**Supplementary Video S1.** **Immuno-****OpenPET-guided surgery for small resectable PC.** The video shows a representative case of immuno-OpenPET-guided surgery with intraperitoneal (ip)-administered ^64^Cu-PCTA-cetuximab in the small resectable orthotopic xPA-1-DC xenograft mouse model at 2 weeks after tumor implantation. Under the immuno-OpenPET-guided surgery, the surgeon detected a 3-mm-sized tumor and resected the tumor while monitoring it with OpenPET real-time imaging. The tumors could not be visually identified by the naked eye. After the surgery, the fluorescence of the tumor was confirmed by stereoscopic fluorescence microscope observation.

**References**

1 Tashima, H. *et al.* Development of a small single-ring OpenPET prototype with a novel transformable architecture. *Phys Med Biol* **61**, 1795-1809 (2016).

2 Tashima, H. *et al.* A single-ring OpenPET enabling PET imaging during radiotherapy. *Phys Med Biol* **57**, 4705-4718 (2012).

3 Tashima, H. *et al.* Real-Time Imaging System for the OpenPET. *IEEE T Nucl Sci* **59**, 40-46 (2012).

4 Hirano, Y. *et al.* Induced radioactivity of a GSO scintillator by secondary fragments in carbon ion therapy and its effects on in-beam OpenPET imaging. *Phys Med Biol* **61**, 4870-4889 (2016).

5 Escuin-Ordinas, H. *et al.* PET imaging to non-invasively study immune activation leading to antitumor responses with a 4-1BB agonistic antibody. *J Immunother Cancer* **1**, 14 (2013).

6 Niccoli, S., Boreham, D. R., Phenix, C. P. & Lees, S. J. Non-radioactive 2-deoxy-2-fluoro-D-glucose inhibits glucose uptake in xenograft tumours and sensitizes HeLa cells to doxorubicin in vitro. *PloS one* **12**, e0187584 (2017).

7 Yoshii, Y. *et al.* Integrated treatment using intraperitoneal radioimmunotherapy and positron emission tomography-guided surgery with ^64^Cu-labeled cetuximab to treat early- and late-phase peritoneal dissemination in human gastrointestinal cancer xenografts. *Oncotarget* **9**, 28935-28950 (2018).
